# Comparative evaluation of handheld vs. high-end ultrasound systems for TIPSS hemodynamic assessment: first impressions

**DOI:** 10.1186/s12880-026-03011-w

**Published:** 2026-11-11

**Authors:** Ivor Dropco, Ursula Vehling-Kaiser, Florian Kaiser, Eleène Kaiser, Sylvia Thurn, Fabian Kück, Wolfgang Herr, Hans Jürgen Schlitt, Christian Stroszczynski, Ernst Michael Jung, Ulrich Kaiser

**Affiliations:** 1 Department of Surgery, University Hospital Regensburg, Regensburg, 93053 Germany; 2 ÜBAG Dr. Vehling-Kaiser MVZ GmbH, Landshut, Germany; 3 Interdisciplinary Ultrasound Center, Department of Radiology, University Hospital Regensburg, Regensburg, Germany; 4 Department of Medical Statistics, University Medical Center Göttingen, Göttingen, Germany; 5 Department of Internal Medicine III, University Hospital Regensburg, Regensburg, Germany

**Keywords:** TIPSS, Ultrasound, Handheld ultrasound, Liver perfusion, Duplex Sonography

## Abstract

**Background:**

Transjugular intrahepatic portosystemic shunt (TIPSS) procedures require reliable post-interventional hemodynamic monitoring to detect shunt dysfunction, for which Doppler ultrasonography is the preferred modality. Owing to the rapid development of handheld ultrasound devices, their diagnostic equivalence to high-end systems warrants investigation.

**Materials and methods:**

A pilot group of 23 patients (*n* = 13 males, 65%; *n* = 7 females; 35%; *n* = 3 unknown) with intrahepatic TIPSS were retrospectively analyzed. Blood flow measurements were obtained and compared using a high-end ultrasound system (Resona R9, Mindray) and modern handheld ultrasound device (Vscan Air™ CL, GE). The TIPSS was evaluated using B-mode and color Doppler sonography. Image quality was assessed using a 5-point scale and flow parameters using statistical analysis. The image quality and the difference between the two devices were analyzed using correlation coefficients and multivariable linear regression models including TIPSS flow values, skin-to-TIPSS distance, and TIPSS dwell time.

**Results:**

Both devices produced clinically interpretable images. However, the high-end device yielded superior image quality in 16 of 23 cases (*p* < 0.001). Significant correlations were observed between the difference in mean TIPSS flow measurements obtained by the two devices (Δ mean TIPSS flow) and corresponding average flow values measured by the two devices (Kendall’s τ = 0.656; *p* < 0.001). Significant associations with Δ TIPSS mean flow were identified for skin-to-TIPSS distance (τ = 0.39; *p* = 0.014) and TIPSS dwell time (τ = −0.39; *p* = 0.017). In multivariable analysis, both mean TIPSS flow measured by the high-end system and skin-to-TIPSS distance remained significantly associated with Δ TIPSS mean flow. The TIPSS mean flow measured by the high-end system correlated with TIPSS dwell time (τ = 0.38; *p* = 0.021). Bland–Altman analysis revealed that handheld measurements tended to overestimate low flows and underestimate high flows.

**Conclusion:**

Handheld ultrasound devices show promising potential for TIPSS surveillance; however, they could lead to distorted flow deviations. Contemporary handheld devices may nevertheless be useful in selected point-of-care settings but cannot yet replace high-end systems for precise hemodynamic monitoring. Larger prospective studies are required.

**Trial registration:**

No clinical trial registration was performed, as this study is a single-center retrospective observational analysis based on existing clinical data. The study did not involve prospective patient enrollment, international procedures, or assignment to treatment groups. Therefore, registration in a clinical trial registry was not applicable.

## Background

The placement of transjugular intrahepatic portosystemic shunt (TIPSS) is an established therapeutic strategy to decrease portal hypertension in patients with complications such as refractory ascites and variceal bleeding [1]. Doppler ultrasound is a cornerstone of TIPSS follow-up because it allows for the noninvasive assessment of shunt flow velocity, patency, and potential stenosis [2, 3]. To date, accurate visualization and quantification of these hemodynamic parameters required high-performance ultrasound systems, which offer superior spatial resolution, color Doppler sensitivity, and customizable presets [4].

Advances in portable imaging have enabled handheld ultrasound devices to be integrated into point-of-care workflows for both adults and pediatric patients [5, 6]. Several studies have demonstrated their diagnostic feasibility for abdominal and vascular applications [7]. Modern handheld ultrasound devices adequately assess abdominal pathologies with clinically acceptable diagnostic agreement compared to high-end systems [8]. These findings indicate that handheld devices serve as viable alternatives to traditional ultrasound systems in specific diagnostic contexts.

Handheld ultrasound poses specific challenges for TIPSS evaluation. TIPSS flow assessment requires precise angle correction, stable Doppler gates, and robust spectral analysis; these features are fully optimized in high-end platforms but only partially implemented in handheld devices [9]. Notably, portable systems exhibit limited Doppler sensitivity, particularly in low-flow states or deeper anatomical regions. Because TIPSS dysfunction can manifest with subtle hemodynamic alterations, even minor measurement inaccuracies may have clinical consequences [10].

To date, there is limited evidence on the reliability of handheld ultrasound for TIPSS monitoring. The pilot dataset evaluated in this study provides one of the earliest direct comparisons between a modern handheld device and a high-end machine in this clinical context. This study aimed to quantify the measurement agreement, identify systematic deviations, and evaluate the practical implications for clinical use.

## Materials and methods

This retrospective pilot study evaluated Doppler-based TIPSS blood flow measurements obtained using two different ultrasound systems: a high-end ultrasound platform (Resona R9, Mindray, 1–6 MHz) and modern handheld ultrasound device (HUD) (Vscan Air™ CL, GE, 2–5 MHz). Examinations were identified through the institutional Picture Archiving and Communication System (PACS) and digital patient management software. TIPSS follow-up examinations were included if both modalities were used in the same patient within a comparable timeframe and if complete Doppler flow documentation was available.

The study was approved by the Ethics Committee of the University of Regensburg (Nr. 23-3491-104) in accordance with the Declaration of Helsinki and the ICH Good Clinical Practice (GPC) guidelines. All participants were informed about the participation, and written informed consent to participate was obtained from all patients.

### Patients

Patients were examined by one experienced physician in our interdisciplinary ultrasound center at the University Hospital between August 2023 and March 2024 and underwent standardized Doppler examination of TIPSS flow measurements. ​ Table 1 shows the patient’s characteristic. Written informed consent was obtained from all individual participants included the study.

### Ultrasound examination

Conventional ultrasound was conducted using a high-end ultrasound system equipped with curved multifrequency transducers (1–6 MHz) and a standardized protocol, followed by imaging with an HUD equipped with a curved multifrequency transducer (2–5 MHz). The patients underwent a complete abdominal examination, and acquired images were digitally archived in PACS.

The patients were examined in the supine position, with the right arm elevated above the head. To ensure adequate TIPSS visualization, imaging was primarily performed intercostally from the patient’s right side. For each examination, B-mode and color-coded Doppler sonography (CCDS) measurements of the TIPSS mid-shunt flow velocity (in cm/s) and shunt diameter (in mm) were extracted, and TIPSS dwell time (in days) and skin-to-TIPSS (in cm) were observed.

The Vscan Air™ CL is a portable, battery-powered ultrasound system featuring a dual-headed probe design. For abdominal and vascular assessment, an integrated curved-array transducer (2–5 MHz; center frequency: 3.3 MHz) was used. The wireless probe communicates with a mobile iOS device via a secure wireless connection, which serves as a display-and-control interface through a dedicated application. The system supports rapid, inductive on-site charging, allowing continuous clinical use. The acquired images were stored anonymously and password-encrypted on the iOS device in accordance with institutional and legal data protection standards.

When using the handheld device, the same examination protocol was followed as for the high-end systems, including B-mode imaging and CCDS with appropriate flow adjustments for portal venous assessment.

### Image rating score

**Fig. 1:**
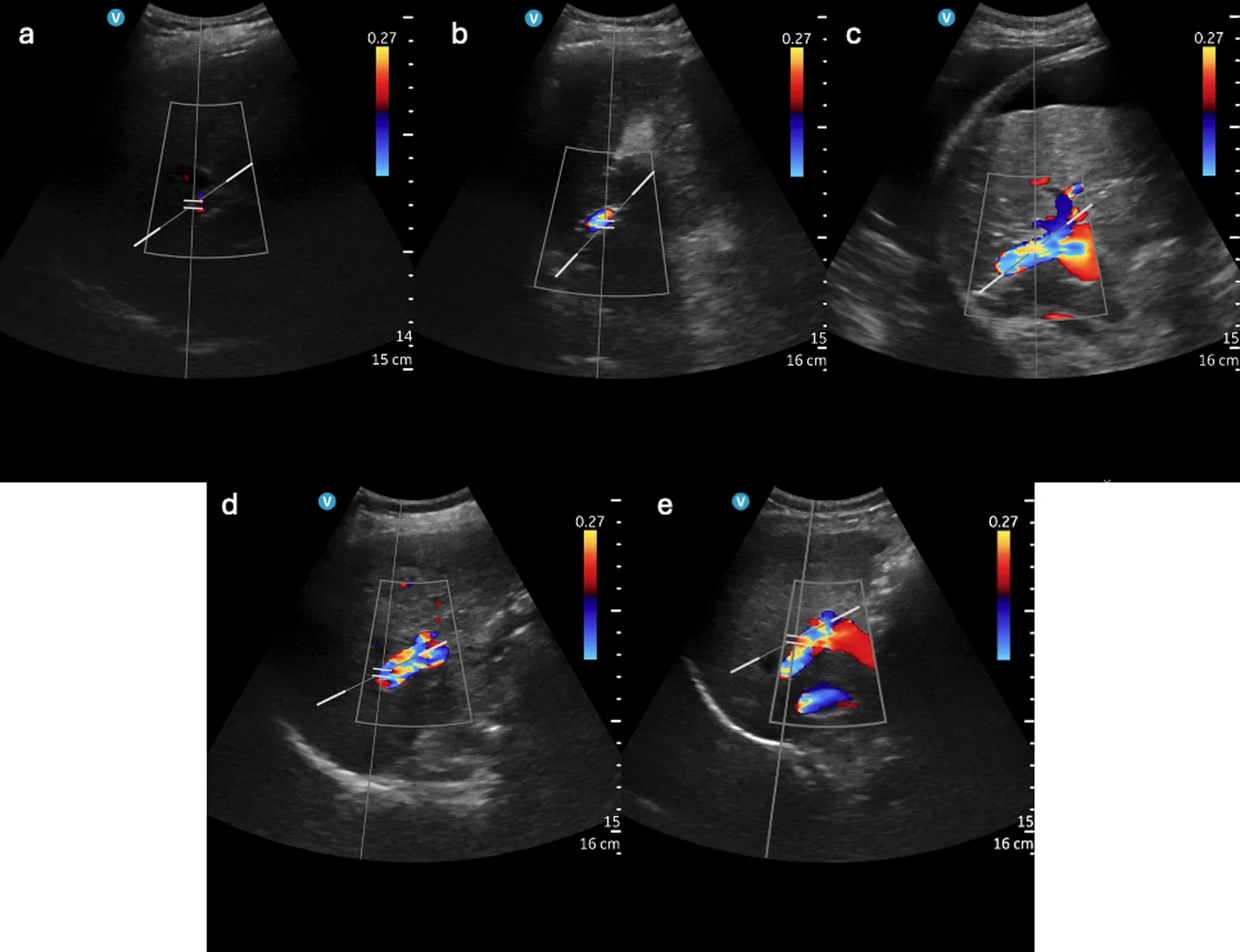
Representative ultrasound images demonstrating the range of image quality observed in the study. Vscan image quality ratings: (**a**) not evaluable, (**b**) hardly evaluable due to artifacts, (**c**) artifact-laden but usable, (**d**) good, and (**e**) excellent image quality

Image quality was scored on a standardized 5-point scale (Fig. 1). The following ratings were possible: 1 = not evaluable, 2 = hardly evaluable due to artifacts, 3 = artifact-laden but usable (e.g. ascites), 4 = good (with minor limitations), and 5 = excellent (without relevant limitations). Two experienced ultrasound examiners independently assessed the acquired images to ensure adequate image quality and their suitability for subsequent analysis. The assessments were performed independently, with each examiner blinded to the other examiner’s evaluation. Furthermore, all data were collected, documented, and stored in accordance with the applicable data protection regulations.

**Fig. 2:**
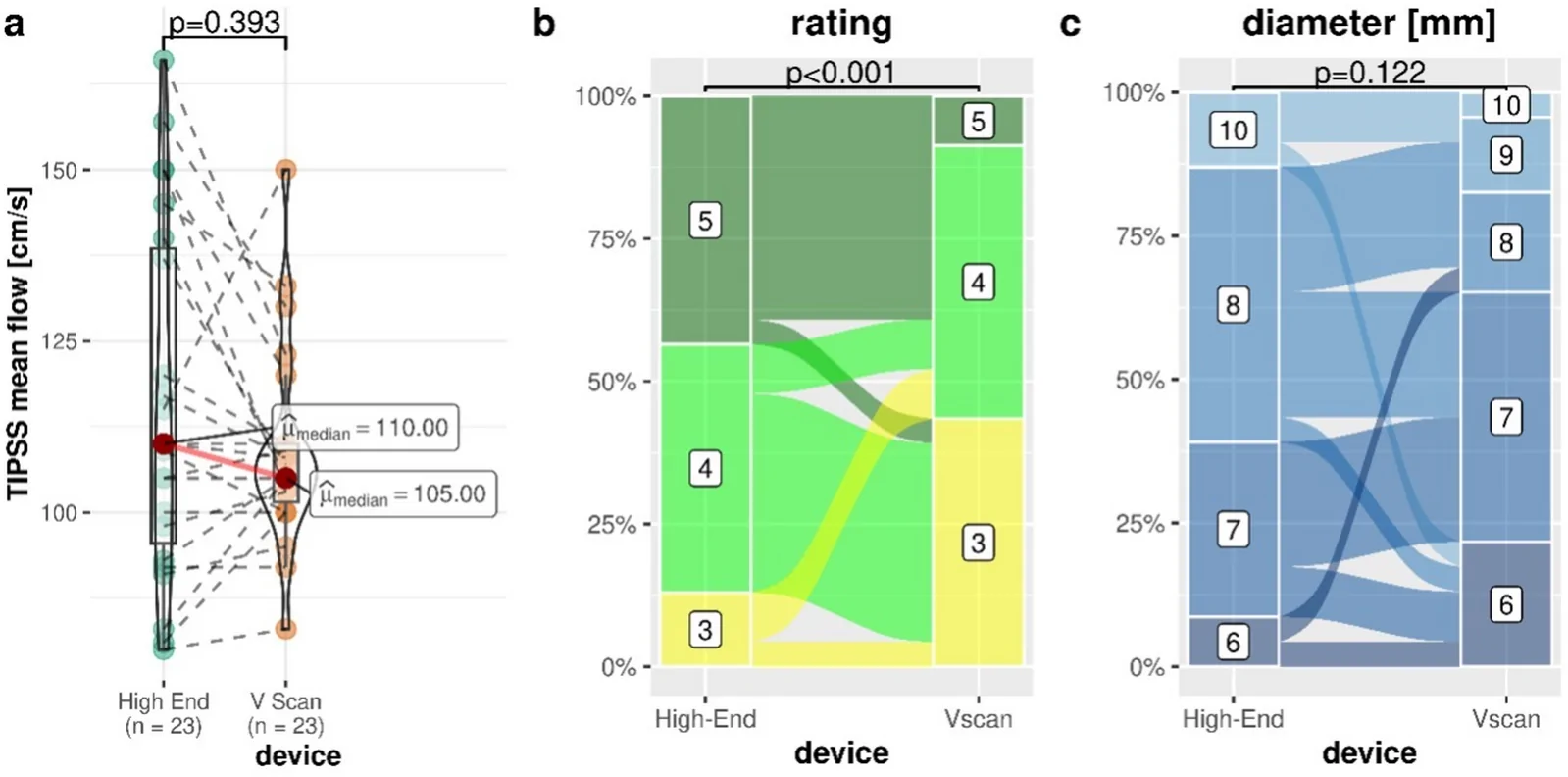
Comparison of TIPSS flow measurements, image quality ratings, and diameter measurements between the high-end and V Scan devices. (**a**) Comparison of TIPSS mean flow velocities measured by the high-end ultrasound system and handheld device (V Scan) using paired data visualization. The median flow values are indicated by highlighted markers, showing a slightly higher median for the high-end system (110 cm/s) compared to the handheld device (105 cm/s). While the overall distributions appear broadly comparable, noticeable variability between paired measurements is evident, as indicated by the spread and direction of the connecting lines. (**b**) Alluvial plot illustrating the distribution and transition of image quality ratings between the high-end ultrasound system and handheld device (V Scan). Image quality was graded on a 5-point scale; the relative proportions of each category are shown for both modalities. For the high-end system, ratings are predominantly concentrated in the higher quality categories (scores 4 and 5), indicating overall superior image quality. In contrast, the handheld device shows a shift toward lower ratings, with a noticeable increase in category 3 and fewer examinations achieving the highest quality score (5). Overall, the figure highlights a systematic reduction in perceived image quality when using the handheld device while demonstrating that most examinations remain within diagnostically acceptable ranges. (**c**) Alluvial plot illustrating the distribution and transition diameter measurements between the high-end ultrasound system and handheld device (V Scan). The distributions appear broadly comparable

**Fig. 3:**
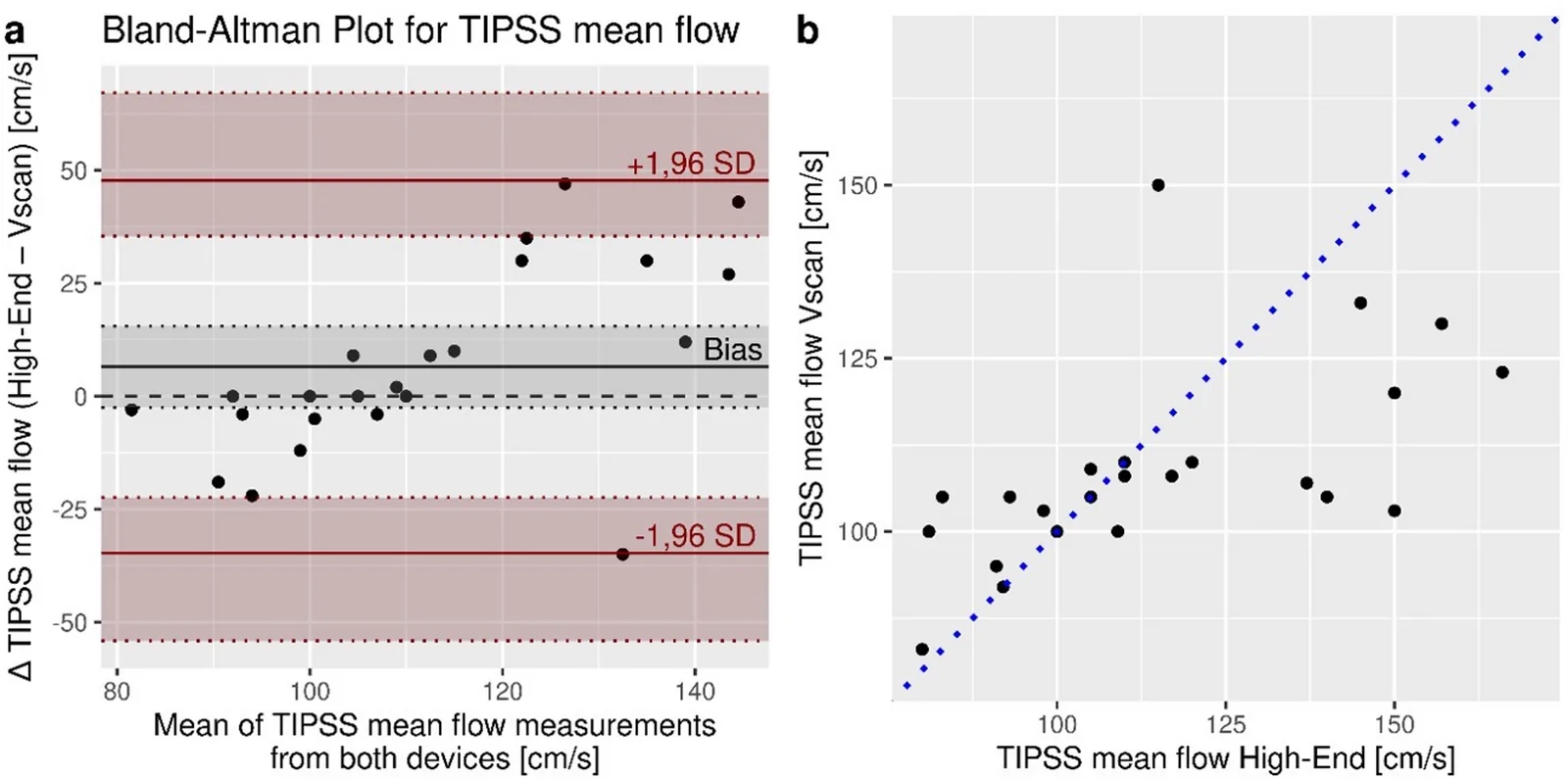
Bland–Altman plot and scatter plot for TIPSS mean flow measured by V Scan and the high-end device. (**a**) Bland–Altman plot illustrating the agreement between handheld and high-end ultrasound measurements of TIPSS flow velocities. The mean difference (bias, black solid line) and limits of agreement (mean ± 1.96 SD, red solid lines) are shown, each with their 95% confidence intervals (shaded areas). Each data point represents a paired measurement, plotted as the difference between the two methods against their mean value. The distribution of points reveals a clear pattern of proportional bias: at lower mean flow velocities, the handheld device tends to yield higher values than the high-end system, while at higher mean flow velocities, it increasingly underestimates flow. This results in a characteristic downward trend across the plot, indicating compression of the measurement range. The mean difference (bias; high-end minus handheld) was 6.5 cm/s, with 95% limits of agreement ranging from − 34.3 to 47.3 cm/s. (**b**) Scatter plot comparing TIPSS mean flow velocities measured with the handheld ultrasound device (V Scan, y-axis) versus the high-end system (x-axis). The blue dotted diagonal line represents the line of identity, indicating perfect agreement between both methods.Most data points cluster around this line, indicating an overall moderate agreement between the two systems. However, a systematic pattern becomes apparent: at lower flow velocities, several points lie above the identity line, indicating that the handheld device tends to overestimate flow compared to the high-end system, while at higher flow velocities, it increasingly underestimates flow

**Fig. 4:**
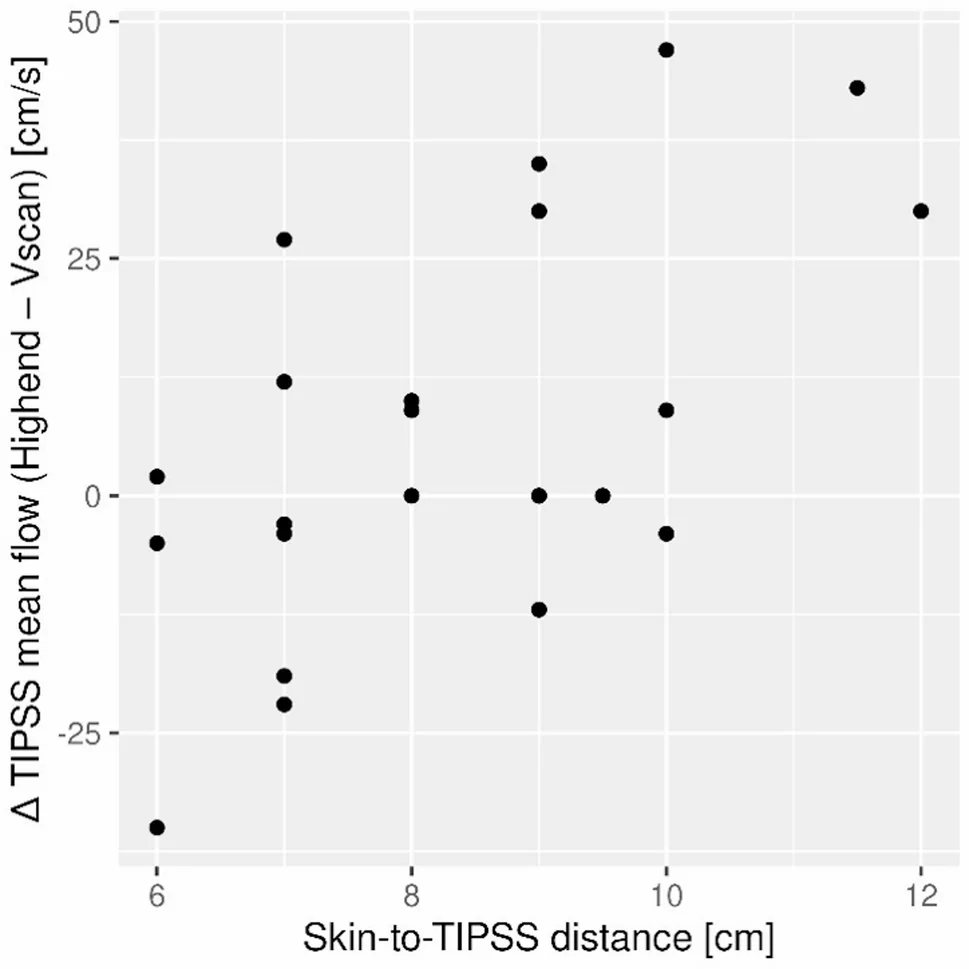
Scatter plot for skin-to-TIPSS distance and the difference in mean TIPSS flow measurements obtained using the two devices (Δ TIPSS mean flow). Scatter plot for skin-to-TIPSS distance (x-axis) and the difference in mean TIPSS flow measurements obtained by using the two devices (Δ TIPSS mean flow, y-axis). A systematic pattern becomes apparent: at higher skin-to-TIPSS distances, the majority of points lie above zero, indicating that the handheld device tends to underestimate flow compared to the high-end system

### Data description

Data are presented as absolute and relative frequencies, mean with standard deviation, median with interquartile range, and minimum and maximum values (Table 1). Image rating score and picture quality are described above.

**Table 1:** Patient characteristics

Parameter	Level	Value
n		23
Age		
	Mean ± SD	55.5 ± 9.5
	median (min; max; [IQR])	59.0 (34.0; 69.0; [50.0; 62.0])
Sex		
	Male	13 (65.0%)
	Female	7 (35.0%)
	Missing	3
Liver quality (cirrhosis, steatohepatitis, Budd–Chiari, homogenously, inhomogeneous)		
	Colombo ARFI	1 (4.8%)
	Inhomogeneous	1 (4.8%)
	Inhomogeneous, atrophy, cirrhosis	1 (4.8%)
	Inhomogeneous, cirrhosis	9 (42.9%)
	Cirrhosis	6 (28.6%)
	Cirrhosis, inhomogeneous	3 (14.3%)
	Missing	2
Ascites		
	Yes	4 (20.0%)
	No	16 (80.0%)
	Missing	3
Ascites length (cm)		
	Mean ± SD	0.590 ± 1.351
	Median (min; max; [IQR])	0.00 (0.00; 4.00; [0.00; 0.00])
	Missing	3
Skin-to-TIPSS distance (cm)		
	Mean ± SD	8.35 ± 1.69
	Median (min; max; [IQR])	8.00 (6.00; 12.0; [7.00; 9.25])
TIPSS dwell time (days)		
	Mean ± SD	1063 ± 654
	Median (min; max; [IQR])	1179 (26.0; 2361; [496; 1532])
	Missing	3
Image quality V Scan [1/2/3/4/5]		
	1	0 (0.0%)
	2	0 (0.0%)
	3	10 (43.5%)
	4	11 (47.8%)
	5	2 (8.7%)
Image quality high-end [1/2/3/4/5]		
	1	0 (0.0%)
	2	0 (0.0%)
	3	3 (13.0%)
	4	10 (43.5%)
	5	10 (43.5%)
TIPSS mean flow V Scan (cm/s)		
	Mean ± SD	109 ± 14
	Median (min; max; [IQR])	105 (83.0; 150; [102; 110])
TIPSS mean flow high-end (cm/s)		
	Mean ± SD	115 ± 26
	Median (min; max; [IQR])	110 (80.0; 166; [95.5; 138])
Diameter V Scan (mm)		
	Mean ± SD	7.37 ± 1.13
	Median (min; max; [IQR])	7.00 (6.00; 10.0; [7.00; 8.00])
Diameter High End (mm)		
	Mean ± SD	7.78 ± 1.09
	Median (min; max; [IQR])	8.00 (6.00; 10.0; [7.00; 8.00])
Diagnosis		
	AIH	1 (4.3%)
	Alcoholic liver cirrhosis	14 (60.9%)
	Budd–Chiari syndrome	1 (4.3%)
	Budd–Chiari syndrome, FNH	1 (4.3%)
	HCC	2 (8.7%)
	NASH	3 (13.0%)
	Primary biliary cholangitis	1 (4.3%)
Δ TIPSS flow		
	Mean ± SD	6.52 ± 20.81
	Median (min; max; [IQR])	0.00 (− 35.0; 47.0; [− 4.00; 19.5])
Δ Diameter		
	Mean ± SD	0.413 ± 1.212
	Median (min; max; [IQR])	0.00 (− 2.50; 4.00; [0.00; 1.00])
|Δ TIPSS mean flow|		
	Mean ± SD	15.6 ± 15.0
	Median (min; max; [IQR])	10.0 (0.00; 47.0; [3.50; 28.5])
|Δ Diameter|		
	Mean ± SD	0.804 ± 0.986
	Median (min; max; [IQR])	1.00 (0.00; 4.00; [0.00; 1.00])

Data are presented as absolute and relative frequencies, mean with standard deviation (SD), median with interquartile range (IQR), and minimum and maximum values. Image quality was scored using a 5-point scale ranging from 1 (not evaluable) to 5 (excellent): the following ratings were possible: 1 = not evaluable, 2 = hardly evaluable due to artifacts, 3 = artifact-laden but usable, 4 = good, and 5 = excellent. AIH: auto-immune hepatitis; FNH: focal nodular hyperplasia; HCC: hepatocellular carcinoma; NASH: non-alcoholic steatohepatitis

### Evaluation of picture quality and statistical analysis

To compare the Vscan and high-end devices, the quality ratings and diameter measurements were visualized using alluvial plots, and Brunner–Munzel tests for paired data were applied. Moreover, the TIPSS flow measurements were analyzed using a Bland–Altman plot.

Associations between various variables were assessed using nonparametric Kendall’s τ correlation coefficients and linear regression models.

For all statistical tests, the significance level was set at α = 5%. Owing to the exploratory nature of this study, no adjustment for multiple testing was applied. Analyses were performed with the statistical programming environment R (version 4.2.3; R Core Team [2023]) using the R packages ggalluvial (version 0.12.5) [11] for alluvial plots, ggstatsplot (version 0.12.4) [12] for box-violin plots, nparcomp (version 3.0) [13] for the Brunner–Munzel test for paired data, and MKinfer (version 1.2) for the Bland–Altman plot.

## Results

Twenty-three patients were included in the analysis (mean age: 55.5 ± 9.5 years; *n* = 13 male, *n* = 7 female, and *n* = 3 unknown; Table 1). The main patient cohort was diagnosed with liver cirrhosis due to alcohol toxicity (*n* = 14). Another patient cohort was diagnosed with hepatocellular carcinoma (*n* = 2) or non-alcoholic steatohepatitis (NASH; *n* = 3). Additionally, one patient each was diagnosed with AIH, Budd–Chiari syndrome, FNH, and primary biliary cholangitis.

No significant differences were found in TIPSS mean flow (*p* = 0.393) or diameter of TIPPS (*p* = 0.122) between the two devices (Fig. 2). In the high-end device, the median TIPPS flow was 110 cm/s, compared to 105 cm/s in the V Scan device (Fig. 2).

Image quality scores were generally within the upper range of the predefined 5-point scale for both ultrasound systems. Nevertheless, the high-end device achieved significantly higher image quality ratings than the handheld device (*p* < 0.001), whereas no examination was rated as non-evaluable (score 1) or hardly evaluable (score 2) (Fig. 2).

Significant correlations were observed between the difference in the mean TIPSS flow measurements obtained by the two devices (Δ TIPSS mean flow) and corresponding average flow values measured by two devices (Kendall’s τ = 0.656; *p* < 0.001; Fig. 3). Additional significant associations were identified for skin-to-TIPSS distance (τ = 0.39; *p* = 0.014; Fig. 4) and TIPSS dwell time (τ = −0.39; *p* = 0.017). Multivariable linear regression analysis confirmed that average TIPSS mean flow measured by the two devices was a significant predictor of Δ TIPSS mean flow (*p* < 0.001; Table 2). Skin-to-TIPSS distance remained significant when adjusting for average mean TIPSS flow (β = 5.6; *p* = 0.009), while TIPSS dwell time was not independently associated with Δ TIPSS mean flow (β = 0.00; *p* = 0.531). In a sensitivity analysis, results were similar when the high-end measurement was used as covariate instead of the average of both devices. Moreover, the TIPSS mean flow measured by the high-end system correlated with TIPSS dwell time (τ = 0.38; *p* = 0.021; Table 3).

**Table 2:** Linear regression models for Δ TIPSS mean flow (number of observations: 20 and 23, respectively). Average TIPSS mean flow was calculated as the arithmetic mean of the measurements from both devices (cm/s)

Model	Variable	β	95% CI	*p*-value
1 2	(Intercept)	−87	(− 139, − 36)	*0.002*
	TIPSS dwell time (days)	0.00	(− 0.01, 0.01)	0.531
	Average TIPSS mean flow (both methods, cm/s)	0.88	(0.48, 1.3)	*< 0.001*
	(Intercept)	−100	(− 146, − 55)	*< 0.001*
	Skin-to-TIPSS distance (cm)	5.6	(1.5, 9.6)	*0.009*
	Average TIPSS mean flow (both methods, cm/s)	0.54	(0.16, 0.91)	*0.007*

Results of Models 1 and 2 including TIPSS dwell time and skin-to-TIPSS distance, which were selected a priori as clinically relevant variables

**Table 3:** Comparison between the specific parameters V Scan and high-end device

Variable	Kendall’s τ	*p*-value
Image quality V Scan, age	−0.14	0.423
Image quality high-end, age	−0.19	0.267
TIPSS mean flow V Scan (cm/s), age	−0.13	0.409
TIPSS mean flow high-end (cm/s), age	0.15	0.340
Diameter V Scan (mm), age	0.08	0.615
Diameter high-end (mm), age	−0.08	0.625
Δ Diameter, age	−0.24	0.147
|Δ Diameter|, age	−0.25	0.153
Δ TIPSS mean flow, age	0.22	0.151
|Δ TIPSS mean flow|, age	0	1.000
Image quality V Scan, skin-to-TIPSS distance (cm)	−0.22	0.217
Image quality high-end, skin-to-TIPSS distance (cm)	−0.27	0.139
TIPSS mean flow V Scan (cm/s), skin-to-TIPSS distance (cm)	0.07	0.665
TIPSS mean flow high-end (cm/s), skin-to-TIPSS distance (cm)	0.28	0.075
Diameter V Scan (mm), skin-to-TIPSS distance (cm)	0.01	0.955
Diameter high-end (mm), skin-to-TIPSS distance (cm)	0.17	0.322
Δ Diameter, skin-to-TIPSS distance (cm)	0.05	0.773
|Δ Diameter|, skin-to-TIPSS distance (cm)	−0.09	0.615
Δ TIPSS mean flow, skin-to-TIPSS distance (cm)	0.39	***0.014***
|Δ TIPSS mean flow|, skin-to-TIPSS distance (cm)	0.1	0.534
Image quality V Scan, TIPSS dwell time (days)	−0.22	0.245
Image quality high-end, TIPSS dwell time (days)	−0.19	0.302
TIPSS mean flow V Scan (cm/s), TIPSS dwell time (days)	−0.17	0.310
TIPSS mean flow high-end (cm/s), TIPSS dwell time (days))	−0.38	***0.021***
Diameter V Scan (mm), TIPSS dwell time (days)	−0.3	0.092
Diameter high-end (mm), TIPSS dwell time (days))	−0.25	0.177
Δ Diameter, TIPSS dwell time (days)	0.08	0.650
|Δ Diameter|, TIPSS dwell time (days)	0.07	0.696
Δ TIPSS mean flow, TIPSS dwell time (days)	−0.39	***0.017***
|Δ TIPSS mean flow|, TIPSS dwell time (days)	−0.19	0.240
Δ TIPSS mean flow, average TIPSS mean flow (both methods, cm/s)	0.656	***< 0.001***
|Δ TIPSS mean flow|, average TIPSS mean flow (both methods, cm/s)	0.43	***0.005***
Δ TIPSS mean flow, TIPSS mean flow V Scan (cm/s)	0.2	0.201
|Δ TIPSS mean flow|, TIPSS mean flow V Scan (cm/s)	0.3	0.055

Δ describes the difference between two measurement techniques (e.g., V Scan vs. high-end ultrasound). |·| denotes the absolute value. Average TIPSS mean flow was calculated as the arithmetic mean of the measurements from both devices (cm/s)

Using Bland–Altman analysis, the association between handheld and high-end ultrasound measurements of TIPSS flow velocities was assessed. Analysis demonstrated a non-uniform distribution of differences, indicating the presence of proportional bias, with the handheld device overestimating lower flow values and underestimating higher flow values. Bland–Altman analysis showed a mean difference (bias; high-end minus handheld) of 6.5 cm/s, with 95% limits of agreement ranging from − 34.3 to 47.3 cm/s. The distribution of differences indicated proportional bias, with differences varying systematically across the range of mean flow velocities (Fig. 3).

Smaller subgroups were too limited in size to allow enough statistical power for more detailed analysis.

## Discussion

Increasing development and progress in handheld ultrasound diagnostics are enabling new applications in everyday clinical practice. Blood flow imaging and its evaluation and interpretation play a critical role. Thus, improvement in small, handheld devices such as Vscan can particularly complete high-end devices measuring TIPSS blood flow.

This study provides crucial evidence on the feasibility, diagnostic strengths, and limitations of HUDs for TIPSS hemodynamic assessment. While handheld ultrasound has rapidly advanced over the last decade and is increasingly used in point-of-care diagnostics for adults and children [5], its application to Doppler-based shunt flow monitoring demonstrates technical challenges. Modern point-of-care devices are commonly used for diagnostics, particularly at the intensive care unit [14].

A key observations in this study is that image quality was consistently adequate with both devices; however, the high-end system outperformed the handheld device in the majority of cases. This is consistent with earlier literature showing that high-end ultrasound platforms offer superior Doppler sensitivity, image penetration, and control over acquisition parameters, which are particularly relevant in deep abdominal vascular structures. The use of high-end systems for quality scoring remains the standard for ultrasound examinations in routine practice [15, 16]. Modern handheld ultrasound devices are capable of producing high-quality images.

Another central finding is the systematic deviation in measured flow velocities between the two devices. The handheld system tended to overestimate low-flow velocities while underestimating higher flows, creating a characteristic compression of the measurement range. This deviation pattern has clinically relevant implications. Low-flow states—often indicative of early shunt stenosis—may be incorrectly interpreted as normal when using a handheld device. Conversely, high flow velocities associated with shunt hyperperfusion may appear deceptively decreased. A greater skin-to-TIPSS distance was associated with larger differences in measured TIPSS mean flow between the handheld and high-end ultrasound systems. As high-end Doppler ultrasound represents the established modality for TIPSS surveillance, these differences suggest that increased tissue depth may decrease the accuracy of the handheld device. Increased tissue depth may contribute to inter-device variability, as Doppler measurements of deep-seated vessels are technically challenging due to ultrasound attenuation and other physical limitations [17, 18]. However, this effect may be more pronounced in handheld ultrasound systems than in high-end systems because of their reduced Doppler sensitivity and the limited availability of advanced acquisition parameter adjustments [19]. Such inaccuracies could delay diagnosis or cause inappropriate management decisions if handheld devices were used as the sole imaging modality [20, 21]. Therefore, further optimization of the Doppler techniques used in handheld devices is crucial. Nevertheless, handheld ultrasound can be used as a fast point-of-care option for physicians. Longer battery life allows clinicians to examine more patients in greater detail and facilitates easier bedside imaging for immobile patients [22, 23].

Moreover, the lack of extensive angle-correction tools and limited Doppler gate customization in handheld platforms may play a role. High-end devices allow fine adjustments to optimize the insonation angle, pulse repetition frequency, gain, and wall-filter settings, while handheld devices often operate under more constrained presets [20].

However, this study also indicates that handheld ultrasound should currently be viewed as a complementary tool rather than a replacement for high-end Doppler evaluation. For definitive TIPSS surveillance, where precise quantification of flow velocities is critical, high-end systems remain the standard. However, the advancements made so far and ongoing progress ensure that handheld devices will become increasingly effective in these areas. Although the observed measurement differences may be clinically relevant when flow velocities approach thresholds suggestive of TIPSS dysfunction, the present study cannot determine whether these deviations would have altered patient management because no invasive hemodynamic or clinical reference standard was available. In clinical practice, measurement differences in suggestive Doppler velocity should always be interpreted in conjunction with additional sonographic or imaging findings and the clinical context [24].

Several developments could help close the performance gap between handheld and high-end systems. Advances in software-based Doppler enhancement, machine-learning-assisted noise suppression, and automated angle correction may significantly improve handheld Doppler accuracy in the coming years. The integration of AI-driven spectral analysis potentially standardizes measurements and reduces operator dependence in handheld ultrasonography [25, 26, 27]. Additionally, prospective multicenter studies with larger cohorts are required to validate the findings of this exploratory pilot analysis and establish clinically significant thresholds for handheld Doppler interpretation in TIPSS patients.

## Conclusion

Although handheld ultrasound technology has advanced rapidly and demonstrated considerable promise for TIPSS monitoring, limitations still remain. High-end ultrasound remains the established modality for TIPSS surveillance and hemodynamic evaluation. Nevertheless, these exploratory findings suggest that contemporary handheld ultrasound devices may represent a promising adjunct for selected point-of-care applications. Future technological advances and further research may help reduce the differences between handheld and high-end ultrasound systems, potentially expanding the role of handheld devices in clinical practice.

## Limitations

The present study has several limitations. In addition to its retrospective design, the relatively small sample size of the analyzed patient cohort (pilot study) limits the statistical power. For image-quality ratings, an additional specific interobserver was not included. Furthermore, the use of devices from different manufacturers restricts the comparability of the results, and the evaluation of only one single high-end ultrasound system and one single handheld device limits the generalizability of the findings to other ultrasound platforms. To draw more nuanced conclusions regarding patients with severe hemodynamic disorders, the sample sizes in future studies must be increased.

## Data Availability

The datasets used and/or analysed during the current study are available from the corresponding author on reasonable request.

## Abbreviations

TIPSS: Transjugular intrahepatic portosystemic shunt
CL: Convex Linear
GE: General Electric
HUD: Handheld ultrasound device
PACS: Picture Archiving and Communication System
CCDS: Color coded doppler sonography
SD: Standard deviation
IQR: Interquartile range
AIH: Auto-immune hepatitis
FNH: Focal nodular hyperplasia
HCC: Hepatocellular carcinoma
NASH: Non-alcoholic steatohepatitis
